# In-hospital death in acute coronary syndrome was related to admission glucose in men but not in women

**DOI:** 10.1186/1475-2840-11-47

**Published:** 2012-05-17

**Authors:** Julio Yoshio Takada, Rogério Bicudo Ramos, Larissa Cardoso Roza, Solange Desiree Avakian, José Antonio Franchini Ramires, Antonio de Pádua Mansur

**Affiliations:** 1 Heart Institute (InCor), University of São Paulo Medical School, Avenue Enéas de Carvalho de Aguiar, 44, 05403-000, São Paulo, Brazil

**Keywords:** Mortality, Myocardial infarction, Hyperglycaemia, Sex, Glycaemia, In-hospital prognosis

## Abstract

**Background:**

Admission hyperglycaemia is associated with mortality in patients with acute coronary syndrome (ACS), but controversy exists whether hyperglycaemia uniformly affects both genders. We evaluated coronary risk factors, gender, hyperglycaemia and their effect on hospital mortality.

**Methods:**

959 ACS patients (363 women and 596 men) were grouped based on glycaemia ≥ or < 200 mg/dL and gender: men with glucose < 200 mg/dL (menG-); women with glucose < 200 mg/dL (womenG-); men with glucose ≥ 200 mg/dL (menG+); and women with glucose ≥ 200 mg/dL (womenG+). A logistic regression analysis compared the relation between gender and glycaemia groups and death, adjusted for coronary risk factors and laboratory data.

**Results group:**

menG- had lower mortality than menG + (OR = 0.172, IC95% 0.062-0.478), and womenG + (OR = 0.275, IC95% 0.090-0.841); womenG- mortality was lower than menG + (OR = 0.230, IC95% 0.074-0.717). No difference was found between menG + vs womenG + (p = 0.461), or womenG- vs womenG + (p = 0.110). Age (OR = 1.067, IC95% 1.031–1.104), EF (OR = 0.942, IC95% 0.915-0.968), and serum creatinine (OR = 1.329, IC95% 1.128-1.566) were other independent factors related to in-hospital death.

**Conclusions:**

Death was greater in hyperglycemic men compared to lower blood glucose men and women groups, but there was no differences between women groups in respect to glycaemia after adjustment for coronary risk factors.

## Introduction

Acute coronary syndromes (ACS) are a set of presentations of coronary artery disease (CAD) and have in common unstable atherosclerotic coronary plaque. Unstable angina (UA), non-ST-elevation myocardial infarction (NSTEMI), and ST-elevation myocardial infarction (STEMI) have differences in treatments and outcomes, but some markers of poor prognosis are common, such as clinical risk scores or several laboratory factors, including C-reactive protein, hyperglycaemia, and elevated brain natriuretic peptide type B [1,2].

Admission blood glucose (ABG) is a risk factor of worse in-hospital and long-term prognosis in ACS [3,4], both in diabetic and nondiabetic patients [5], but its influence on mortality can be different along time after an acute event [6,7], and according to the diagnosis of patients admitted to intensive care units [8]. In ACS patients, women have more risk factors for CAD and are older than men, so after adjustment for confounders, this difference disappears, with the exception of STEMI patients when women still have higher mortality than men [9-11]. Champney et al [12] reported that at ages 50 to 70 years, women with STEMI do have an increased adjusted mortality risk in comparison with men, but not in other age ranges. On the other hand, different from that observed in men, ABG had a non-uniform relationship with ACS mortality in women, as described by Cubbon et al [13], who found a proportional increase in the hazard ratio for mortality based on the interaction between sex and ABG with an increase in glucose levels in men. Women however did not have a greater risk of mortality at levels of glucose >11.1 mmol/L (200 mg/dL).

This study evaluated the influence of ABG on hospital mortality in ACS and its subgroups of presentations, in particular whether this influence occurs in women in the same way that it occurs in men.

## Methods

### Study design and population

Patients with ACS admitted consecutively between January 2004 and April 2007 to an urban academic cardiology emergency single-center in São Paulo, Brazil, were prospectively evaluated. The Ethics Committee of the hospital approved this study and written informed consent was obtained from all participants. Baseline clinical and admission laboratory characteristics, CAD risk factors, in-hospital mortality, and treatments were observed. Ejection fraction was obtained by echocardiography at the second day of hospital stay. A total of 1304 ACS patients were included, and a subgroup of 959 patients had complete data to be studied. Inclusion criteria were ACS hospital admission, according to the international consensus definition, age older than 18 years, and willing to provide written informed consent. Exclusion criteria were incomplete laboratory data. A sample size of at least 118 patients, with 59 subjects per treatment group, was determined to give 80% power to detect a 6% difference in the incidence of cardiovascular events with a 5% significance level.

### Definitions and data collection

Clinical outcome was defined as death. Because 90% of patients at our emergency department undergo angiographic study in 48 hours, reinfarction or refractory angina are infrequent events at hospitalization. Blood samples were collected at admission to the emergency department and ejection fraction was obtained at second day of stay by echocardiography with the biplane Simpson's method. We selected the level of hyperglycaemia above 200 mg/dL to separate the highest stratum seen in the literature [13] for admission nonfasting blood glucose.

Patients were grouped by gender and ABG below (“moderate hyperglycaemia” = G-) or above (“higher hyperglycaemia” = G+) 200 mg/dL: menG- = men with glucose ≤ 200 mg/dL; womenG- = women with glucose ≤ 200 mg/dL; menG + = men with glucose ≥ 200 mg/dL; and womenG + = women with glucose ≥ 200 mg/dL. We observed patient’s distribution in acute coronary syndromes. UA was defined by hospital admission with thoracic pain (or any equivalent) considered to be of myocardial ischemic origin, and non ST-elevation on electrocardiographic or myocardial necrosis marker changes at first consultation. NSTEMI was defined by symptoms of myocardial ischemic origin, myocardial necrosis marker elevation, and no ST-elevation at first electrocardiogram in the emergency department. STEMI was considered when symptoms of myocardial ischemic origin and ST-elevation segment on electrocardiogram were present [14]. ABG was managed based on emergency physicians routine: subcutaneous intermittent insulin doses until blood glucose below 140 mg/dL, and point-of-care glucose control after first blood glucose measurement.

Treatments were defined as medical therapy only, when patients were not candidate to angiography or the invasive study did not result in surgical or percutaneous intervention, and percutaneous (angioplasty) or emergency coronary surgery.

### Statistical analysis

All data are described as rates and frequencies or means with standard deviations, as appropriate. Differences in the distribution of select characteristics between patient groups were examined using the chi-square test and Fisher’s exact test for categorical variables. The analysis was performed using the Student *t* test for normally distributed continuous variables and the Mann–Whitney and Kruskal-Wallis tests for nonparametric variables. General linear models analyzed the hierarchical differences of 3 groups, instead of ANOVA of the NPARWAY1 approach. We built an adjusted model in multivariate regression to analyze the independent variables associated with in-hospital death: age, family history of CAD, prior coronary artery disease, hypertension, smoking, diabetes, categorical admission blood glucose < or ≥ 200 mg/dL, gender, ejection fraction and serum creatinine. This analysis was done for all ACS patients and for AU, NSTEMI, and STEMI patients. In ACS analysis, we added type of ACS presentation to the logistic model. Sex and categorical blood glucose were analyzed as a specific subgroup in the logistic model. We performed analysis by the stepwise approach, and variables with p value ≤ 0.10 were included in the model. Alternatively, we performed an interaction analysis of gender, categorical admission blood glucose < or ≥ 200 mg/dL and type of ACS presentation. Two-sided p values < 0.05 were considered statistically significant. All statistical analyses were performed using SAS software version 9.2 for Windows (SAS Institute Inc., Cary, NC, USA).

## Results

Table 1 shows a comparison between types of ACS. Proportion of males increased from UA to NSTEMI (53.9% vs 63.2%, p = 0.010), UA to STEMI (53.9% vs 71.7%, p < 0.001), and NSTEMI to STEMI (63.2% vs 71.7%, p = 0.035). STEMI patients were younger than NSTEMI (p < 0.001), had lower prior coronary artery disease than NSTEMI or UA (p < 0.001), hypertension (p < 0.001), dyslipidemia (p < 0.001), diabetes (p = 0.003), total cholesterol (p = 0.009), ejection fraction (p < 0.001 in comparison with UA); more of them were current smokers (p < 0.001) than UA or NSTEMI patients.

**Table 1:** Univariate clinical and demographic comparison between unstable angina, non-ST elevation and ST-elevation myocardial infarction patients

	UA*	**NSTEMI**	**STEMI**	**p-value†**
Patients, n (%)	304 (31.7)	443 (46.2)	212 (22.1)	
Male, n (%)	164 (53.9)	280 (63.2)	152 (71.7)	a, b, c
Age (years) ‡	60.7 ± 0.4	63.3 ± 11.9	59.2 ± 12.8	a, c
Prior coronary artery disease, n (%)	198 (65.1)	239 (53.9)	70 (33.0)	a, b, c
Hypertension, n (%)	258 (84.9)	365 (82.4)	146 (68.9)	b, c
Current smoker, n (%)	51 (16.8)	89 (20.1)	72 (34.0)	b, c
Dyslipidemia, n (%)	171 (56.2)	228 (51.5)	67 (31.6)	b, c
Diabetes mellitus, n (%)	104 (34.2)	167 (37.7)	55 (25.9)	c
Family history of coronary artery disease, n (%)	44 (14.5)	84 (18.9)	35 (16.5)	Non-significant
Hemoglobin (g/dL)	13.7 ± 1.6	13.6 ± 1.9	14.2 ± 1.8	b, c
Serum creatinine (mg/dL)	1.2 ± 1.0	1.3 ± 1.3	1.1 ± 0.6	a
Troponin I (ng/mL)	1.3 ± 6.5	20.6 ± 34.5	56.6 ± 57.9	a, b, c
Cholesterol (mg/dL)	185.6 ± 48.3	180.5 ± 49.0	173.5 ± 44.0	b
Admission glucose (mg/dL)	126.9 ± 60.4	136.7 ± 72.1	136.4 ± 66.7	Non-significant
Ejection fraction (%)†	56.6 ± 14.0	51.2 ± 15.5	49.4 ± 12.9	a, b
Angiography, n (%)	289 (95.1)	421 (95.0)	212 (100.0)	b, c
Medical therapy only, n (%)	138 (45.4)	147 (33.2)	23 (10.8)	a, b, c
Percutaneous coronary intervention, n (%)	145 (47.7)	263 (59.4)	177 (83.5)	a, b, c
Surgery, n (%)	21 (6.9)	35 (7.9)	14 (6.6)	Non-significant
Length of stay (days)	4.6 ± 8.1	7.3 ± 12.3	4.8 ± 5.8	a, c
Mortality, n (%)	5 (1.6)	22 (5.0)	8 (3.8)	a

*UA = unstable angina; NSTEMI = non-ST elevation myocardial infarction; STEMI = ST elevation myocardial infarction; †p-value: a = UA versus NSTEMI significant; b = UA versus STEMI significant; c = NSTEMI versus STEMI significant; ‡continuous variables are shown as mean (standard deviation), and categorical variables are shown as number (percentage).

Of all patients (959), men totaled 596 and women 363 (UA 304, NSTEMI 443, and STEMI 212 patients). In STEMI patients, only 12 were treated with thrombolytic drugs and 200 with primary angioplasty. Table 2 presents clinical and demographic patient characteristics of all ACS, UA, NSTEMI and STEMI patients, grouped by ABG < or ≥ 200 mg/dL and gender. Univariate analysis showed that higher ABG patients were older (p < 0.001), had more hypertension (p < 0.001), diabetes (p < 0.001), dyslipidemia (p = 0.002), higher mortality (p < 0.001) and length of stay (p = 0.003), less smoking (p = 0.008) and lower hemoglobin levels (p < 0.001), needed more surgical interventions (p = 0.046), and fewer percutaneous coronary interventions (p = 0.038). Men had higher troponin (p = 0.003) and hemoglobin levels (p < 0.001), but lower total cholesterol (p < 0.001) and ejection fraction (p < 0.001) than women had. In unstable angina, higher ABG patients had more prior CAD (p = 0.013), hypertension (p = 0.019), and diabetes (p < 0.001). In NSTEMI, higher ABG patients were older (p = 0.006), had more hypertension (p = 0.026), diabetes (p < 0.001), higher length of stay (p = 0.002), mortality (p < 0.001), lower ejection fraction (p < 0.001), angiography (p = 0.028), and percutaneous coronary intervention (p = 0.006). In STEMI, higher ABG patients had more hypertension (p = 0.009), diabetes (p < 0.001), mortality (p = 0.012), and less smoking (p = 0.036).

**Table 2:** Clinical and demographic characteristics of acute coronary syndrome, unstable angina, non-ST elevation, and ST-elevation myocardial infarction patients, grouped by gender and glycaemia glucose < or ≥ 200 mg/dL

ACS*	**Men glucose <200**	**Women glucose <200**	**Men glucose ≥200**	**Women glucose ≥200**	**p** ††
Patients	454 (47.3)	242 (25.2)	142 (14.8)	121 (12.6)	
Age (years) †	60.1 ± 11.4	61.9 ± 11.7	63.1 ± 11.3	64.6 ± 12.9	<0.001
Prior coronary artery disease, n (%)	244 (53.7)	113 (46.7)	81 (57.0)	69 (57.0)	0,129
Hypertension, n (%)	338 (74.4)	197 (81.4)	122 (85.9)	112 (92.6)	<0.001
Current smoker, n (%)	119 (26.2)	53 (21.9)	23 (16.2)	17 (14.0)	0,008
Dyslipidemia, n (%)	192 (42.3)	133 (55.0)	73 (51.4)	68 (56.2)	0,002
Diabetes mellitus, n (%)	80 (17.6)	50 (20.7)	103 (72.5)	93 (76.9)	<0.001
Family history of coronary artery disease, n (%)	77 (17.0)	40 (16.5)	23 (16.2)	23 (19.0)	0,929
Hemoglobin (g/dL)	14.3 ± 1.8	13.2 ± 1.5	13.8 ± 1.9	12.9 ± 1.8	<0.001
Serum creatinine (mg/dL)	1.2 ± 0.8	1.1 ± 1.3	1.3 ± 1.0	1.3 ± 1.4	0,331
Troponin I (ng/mL)	25.4 ± 44.4	14.8 ± 32.9	25.0 ± 44.7	16.6 ± 31.8	0,003
Cholesterol (mg/dL)	175.6 ± 44.6	194.5 ± 54.3	166.5 ± 43.9	188.6 ± 43.1	<0.001
Admission glucose (mg/dL)	101.6 ± 15.8	100.0 ± 14.9	211.2 ± 67.7	229.0 ± 84.1	<0.001
Ejection fraction (%)	50.9 ± 14.2	58.2 ± 13.3	46.8 ± 14.9	51.9 ± 15.6	<0.001
Angiography, n (%)	445 (98.0)	229 (94.6)	133 (93.7)	115 (95.0)	0,036
Medical therapy only, n (%)	134 (29.5)	88 (36.4)	42 (29.6)	44 (36.4)	0,184
Percutaneous coronary intervention, n (%)	298 (65.6)	137 (56.6)	84 (59.2)	66 (54.6)	0,038
Surgery, n (%)	23 (5.1)	19 (7.8)	16 (11.3)	12 (9.9)	0,046
Length of stay (days)	5.0 ± 6.6	5.7 ± 10.5	8.2 ± 16.1	7.2 ± 9.6	0,003
Mortality, n (%)	6 (1.3)	4 (1.6)	15 (10.6)	10 (8.3)	<0.001
**UA**					
Patients, n (%)	132 (43.4)	100 (32.9)	32 (10.5)	40 (13.2)	
Age (years)*	59.9 ± 10.9	60.7 ± 9.6	61.6 ± 10.7	62.5 ± 10.8	0.537
Prior coronary artery disease, n (%)	91 (68.9)	55 (55.0)	27 (84.4)	25 (62.5)	0.013
Hypertension, n (%)	103 (78.0)	88 (88.0)	31 (96.9)	36 (90.0)	0.019
Current smoker, n (%)	25 (18.9)	16 (16.0)	6 (18.7)	4 (10.0)	0.595
Dyslipidemia, n (%)	68 (51.5)	65 (65.0)	17 (53.1)	21 (52.5)	0.197
Diabetes mellitus, n (%)	27 (20.5)	23 (23.0)	24 (75.0)	30 (75.0)	<0.001
Family history of coronary artery disease, n (%)	22 (16.7)	14 (14.0)	4 (12.5)	4 (10.0)	0.734
Hemoglobin (g/dL)	14.1 ± 1.7	13.3 ± 1.4	13.9 ± 1.9	13.5 ± 1.5	0.002
Serum creatinine (mg/dL)	1.3 ± 1.2	0.9 ± 0.3	1.2 ± 0.5	1.3 ± 1.5	0.032
Troponin I (ng/mL)	1.0 ± 3.7	0.5 ± 1.6	1.2 ± 3.5	3.7 ± 16.0	0.067
Cholesterol (mg/dL)	176.3 ± 44.1	193.9 ± 46.7	178.6 ± 63.5	200.5 ± 48.9	0.017
Admission glucose (mg/dL)	99.6 (14.6)	99.3 (15.4)	222.5 (66.3)	209.5 (67.3)	<0.001
Ejection fraction (%)†	56.1 ± 13.7	59.5 ± 12.8	50.7 ± 15.5	55.5 ± 15.2	0.069
Angiography, n (%)	127 (96.2)	93 (93.0)	30 (93.8)	39 (97.5)	0.593
Medical therapy only, n (%)	57 (43.2)	53 (53.0)	11 (34.4)	17 (42.5)	0.231
Percutaneous coronary intervention, n (%)	67 (50.8)	40 (40.0)	17 (53.1)	21 (52.5)	0.306
Surgery, n (%)	8 (6.1)	7 (7.0)	4 (12.5)	2 (5.0)	0.587
Length of stay (days)	4.9 ± 9.1	3.5 ± 3.2	7.3 ± 14.6	4.2 ± 4.9	0.115
Mortality, n (%)	1 (0.8)	0	4 (12.5)	0	<0.001
**NSTEMI**					
Patients, n (%)	208 (46.9)	103 (23.3)	72 (16.3)	60 (13.5)	
Age (years)*	61.2 ± 11.4	64.4 ± 12.9	65.7 ± 10.3	65.7 ± 12.5	0.006
Prior coronary artery disease, n (%)	115 (55.3)	46 (44.7)	42 (58.3)	36 (60.0)	0.159
Hypertension, n (%)	161 (77.4)	85 (82.5)	64 (88.9)	55 (91.7)	0.026
Current smoker, n (%)	50 (24.0)	21 (20.4)	7 (9.7)	11 (18.3)	0.073
Dyslipidemia, n (%)	97 (46.6)	53 (51.5)	41 (56.9)	37 (61.70	0.151
Diabetes mellitus, n (%)	41 (19.7)	23 (22.3)	55 (76.4)	48 (80.0)	<0.001
Family history of coronary artery disease, n (%)	37 (17.8)	19 (18.4)	14 (19.4)	14 (23.3)	0.811
Hemoglobin (g/dL)	14.3 ± 1.8	13.0 ± 1.6	13.2 ± 2.0	12.7 ± 1.9	<0.001
Serum creatinine (mg/dL)	1.2 ± 0.7	1.3 ± 1.9	1.5 ± 1.4	1.4 ± 1.5	0.586
Troponin I (ng/mL)	22.6 ± 36.9	18.4 ± 34.4	19.5 ± 31.2	18.9 ± 29.7	0.734
Cholesterol (mg/dL)	178.4 ± 47.2	194.5 ± 61.2	167.1 ± 38.0	182.6 ± 39.8	0.011
Admission glucose (mg/dL)	100.8 ± 16.5	101.9 ± 14.8	202.7 ± 62.6	241.3 ± 98.7	<0.001
Ejection fraction (%)†	49.8 ± 14.9	59.3 ± 13.5	44.7 ± 14.9	49.0 ± 16.0	<0.001
Angiography, n (%)	204 (98.1)	97 (94.2)	65 (90.3)	55 (91.7)	0.028
Medical therapy only, n (%)	63 (30.3)	31 (30.1)	27 (37.5)	26 (43.3)	0.202
Percutaneous coronary intervention, n (%)	135 (64.9)	66 (64.1)	36 (50.0)	26 (43.3)	0.006
Surgery, n (%)	10 (4.8)	8 (7.8)	9 (12.5)	8 (13.3)	0.064
Length of stay (days)	5.1 ± 5.2	7.7 ± 14.5	10.7 ± 20.1	9.9 ± 12.4	0.002
Mortality, n (%)	4 (1.9)	3 (2.9)	8 (11.1)	7 (11.7)	<0.001
**STEMI**					
Patients, n (%)	114 (53.8)	39 (18.4)	38 (17.9)	21 (9.9)	
Age (years)*	58.1 ± 11.9	58.7 ± 12.3	59.3 ± 12.5	65.7 ± 17.1	0.096
Prior coronary artery disease, n (%)	38 (33.3)	12 (30.8)	12 (31.6)	8 (38.1)	0.945
Hypertension, n (%)	74 (64.9)	24 (61.5)	27 (71.1)	21 (100)	0.009
Current smoker, n (%)	44 (38.6)	16 (41.0)	10 (26.3)	2 (9.5)	0.036
Dyslipidemia, n (%)	27 (23.7)	15 (38.5)	15 (39.5)	10 (47.6)	0.052
Diabetes mellitus, n (%)	12 (10.5)	4 (10.3)	24 (63.2)	15 (71.4)	<0.001
Family history of coronary artery disease, n (%)	18 (15.8)	7 (17.9)	5 (13.2)	5 (23.8)	0.748
Hemoglobin (g/dL)	14.5 ± 1.8	13.2 ± 1.6	14.8 ± 1.4	12.7 ± 2.1	<0.001
Serum creatinine (mg/dL)	1.2 ± 0.4	1.1 ± 1.0	1.1 ± 0.4	1.1 ± 0.5	0.758
Troponin I (ng/mL)	58.8 ± 59.4	41.0 ± 48.7	56.1 ± 65.3	34.7 ± 47.8	0.173
Cholesterol (mg/dL)	169.3 ± 39.4	195.9 ± 56.6	156.5 ± 34.7	180.7 ± 36.1	0.001
Admission glucose (mg/dL)	105.4 ± 15.4	96.7 ± 13.6	217.9 ± 77.2	230.9 ± 60.3	<0.001
Ejection fraction (%)†	47.9 ± 12.0	51.5 ± 12.1	47.9 ± 14.6	55.4 ± 13.8	0.098
Angiography, n (%)	114 (100.0)	39 (100.0)	38 (100.0)	21 (100.0)	1.000
Medical therapy only, n (%)	14 (12.3)	4 (10.3)	4 (10.5)	1 (4.8)	0.786
Percutaneous coronary intervention, n (%)	96 (84.2)	31 (79.5)	31 (81.6)	19 (90.5)	0.719
Surgery, n (%)	5 (4.4)	4 (10.3)	3 (7.9)	2 (9.5)	0.543
Length of stay (days)	4.6 ± 5.3	5.9 ± 9.2	4.2 ± 3.4	4.9 ± 2.8	0.546
Mortality, n (%)	1 (0.9)	1 (2.6)	3 (7.9)	3 (14.3)	0.012

*ACS = acute coronary syndrome; UA = unstable angina; NSTEMI = non-ST elevation myocardial infarction; STEMI = ST elevation myocardial infarction; †continuous variables are shown as mean (standard deviation), and categorical variables are shown as number (percentage); †† *p-values* refers to four-groups comparisons.

**Figure 1:**
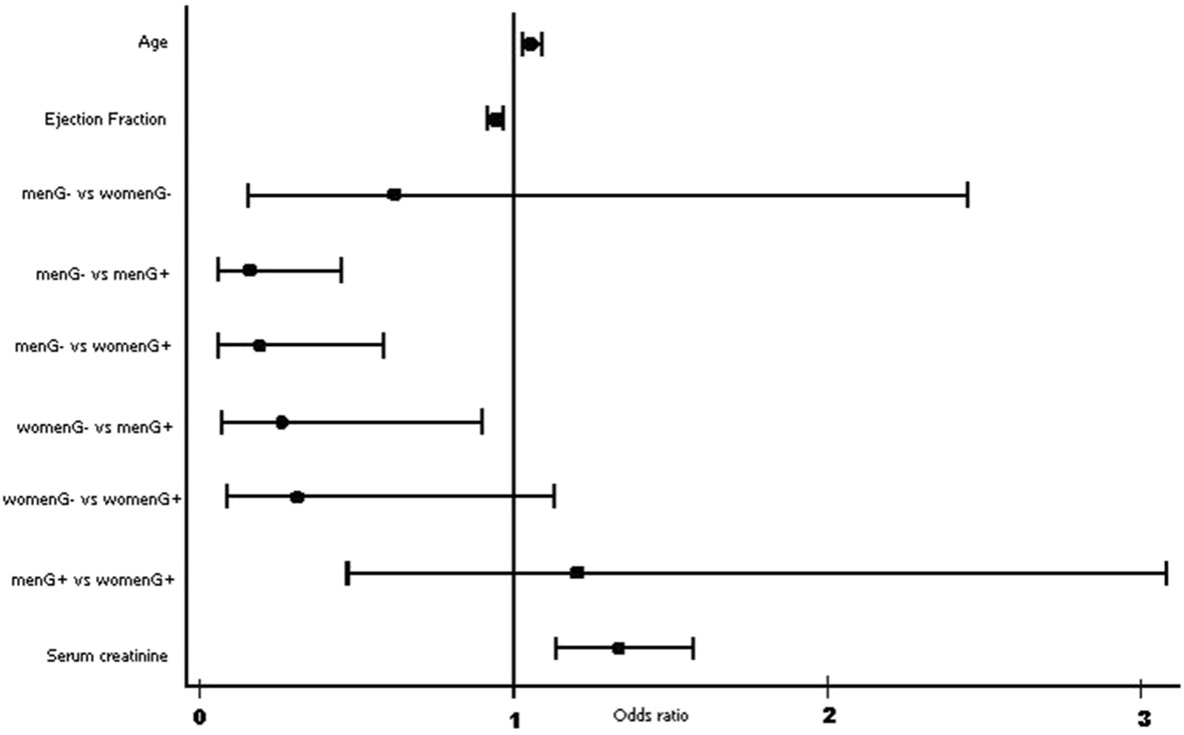
Odds ratio of in-hospital adjusted mortality of groups gender and hyperglycaemia; menG- = men with moderate hyperglycaemia (< 200 mg/dL); womenG- = Women with moderate hyperglycaemia (< 200 mg/dL); menG + = Men with higher hyperglycaemia (≥ 200 mg/dL); womenG + = Women with higher hyperglycaemia (≥ 200 mg/dL).

In multivariate logistic analysis, the adjusted model showed as independent factors related to ACS in-hospital death, age (OR = 1.057, IC 95% 1.022 – 1.093; p = 0.001), serum creatinine level (OR = 1.337, IC 95% 1.335 – 1.574; p < 0.001), ejection fraction (OR = 0.943, IC 95% 0.917 – 0.970; p < 0.001), as showed in Figure 1. When interaction between gender and categorical admission blood glucose was adjusted by logistic model, womenG- had lower mortality than menG + (OR 0.259, IC 95% 0.074 – 0.905; p = 0.034), but no difference with womenG + (OR 0.312, IC 95% 0.086 – 1.130; p = 0.076). Group menG- had lower mortality than menG + (OR 0.160, IC 95% 0.057 – 0.452; p < 0.001) or womenG + (OR 0.193, IC 95% 0.063 – 0.588; p = 0.004). There was no difference between gender in admission blood glucose < 200 mg/dL (OR 0.618, IC 95% 0.0156 – 2.449; p = 0.494) or ≥ 200 mg/dL (OR 1.202, IC 95% 0.470 – 3.075; p = 0.701).

When patients were separated by ACS type, in UA group we observed that age (OR 1.168, IC 95% 1.024 – 1.332; p = 0.021) and admission blood glucose (OR 1.019, IC 95% 1.007 – 1.031; p = 0.002) were independent factors related to in-hospital death, whereas in NSTEMI we had age (OR 1.090, IC 95% 1.038 – 1.144; p < 0.001), ejection fraction (OR 0.942, IC 95% 0.908 – 0.977; p = 0.001), creatinine (OR 1.357, IC 95% 1.124 – 1.639; p = 0.001), and admission blood glucose (OR 1.005, IC 95% 1.000 – 1.009; p = 0.037). Finally, in the STEMI group we found creatinine (OR 13.446, IC 95% 2.840 – 63.656; p = 0.001), and female gender (OR 0.147, IC 95% 0.024 – 0.886; p = 0.036 for men versus women) as independent risks for death, as showed in Table 3.

**Table 3:** Odds ratio of in-hospital adjusted mortality for unstable angina, non-ST elevation myocardial infarction, and ST elevation myocardial infarction

UA*	**Odds Ratio**	**Inferior limit 95% IC**	**Superior limit 95% IC**	**p**
Age	1.168	1.024	1.332	0.021
Admission blood glucose	1.019	1.007	1.031	0.002
**NSTEMI**				
Age	1.090	1.038	1.144	<0.001
Ejection fraction	0.942	0.908	0.977	0.001
Creatinine	1.357	1.124	1.639	0.001
Admission blood glucose	1.005	1.000	1.009	0.037
**STEMI**				
Ejection fraction	0.939	0.878	1.005	0.069
Creatinine	13.446	2.840	63.656	0.001
Men vs Women	0.147	0.024	0.886	0.036

* UA = unstable angina; NSTEMI = non-ST elevation myocardial infarction; STEMI = ST elevation myocardial infarction; IC = interval of confidence.

**Figure 2:**
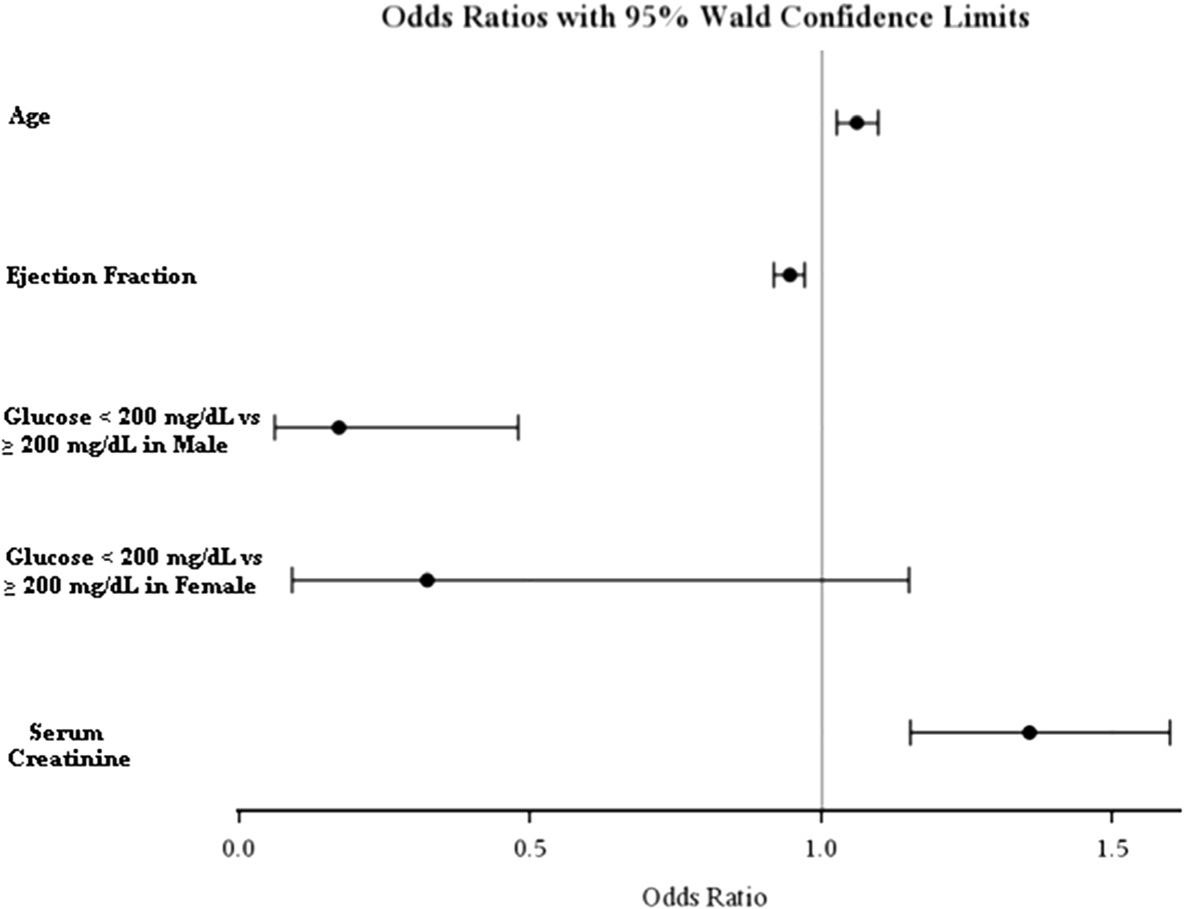
Odds ratio of in-hospital adjusted mortality of interaction between gender and hyperglycaemia.

In interaction analysis, multivariate logistic analysis model showed age (OR 1.060, IC 95% 1.025 - 1.096; p < 0.001), ejection fraction (OR 0.944, IC 95% 0.918 - 0.971; p < 0.001), serum creatinine (OR 1.356, IC 95% 1.151 - 1.598; p < 0.001) and interaction ABG < 200 mg/dL in male gender (OR 0.171, IC 95% 0.061 - 0.478; p = 0.017 in comparison to ABG ≥ 200 mg/dL). In female gender, categorical blood glucose < or ≥ 200 mg/dL had no difference (OR 0.321, IC 95% 0.090 - 1.151; p = 0.078). Figure 2 shows interaction between gender and categorical blood glucose group.

## Discussion

The results of this study show that ABG influences men’s in-hospital mortality from ACS, but this effect was not observed in women when patients of the same gender and different degrees of ABG were compared. According to ACS type of presentation, ABG was an independent factor related to in-hospital mortality in UA and NSTEMI, but not with STEMI in this study population. In fact, differences between diagnostic categories can exist, like that found in the Falciglia et al study [8], a significant association between hyperglycaemia and adjusted mortality was seen in unstable angina, acute myocardial infarction, congestive heart failure, arrhythmia, ischemic and hemorrhagic stroke, gastrointestinal bleeding, acute renal failure, pneumonia, pulmonary embolism and sepsis, but not in patients with chronic obstructive pulmonary disease, hepatic failure, and gastrointestinal neoplasm, or those admitted to the intensive care unit after surgery for coronary artery bypass graft, peripheral vascular disease, and hip fracture. But in our study, ABG was not an independent factor in STEMI, and this is different from other studies [4,7]. Lower age of STEMI patients in our study could explain both the similarity in mortality between STEMI and NSTEMI groups and the absence of the influence of glucose on STEMI mortality.

Several hypotheses have been raised to explain the role of hyperglycaemia in the worse prognosis in ACS. First, ABG could reflect uncontrolled or undiagnosed diabetes and a more advanced atherosclerosis. To justify this point of view, there is an association of ABG with elevation of hemoglobin A1C in these patients [15,16]. Second, ABG is a common acute adrenergic signal of stress and is present in myocardial infarction [17], as well as other severe acute illnesses [8], whereas increased catecholamine levels result in decreased insulin secretion and increased insulin resistance [18]. Third, several studies show that hyperglycaemia could by itself be harmful to ischemic myocardium. For example, acute increases in plasma glucose levels have significant hemodynamic effects, even in normal subjects. In one study, maintenance of plasma glucose levels at 15.0 mmol/L (270 mg/dL) for 2 hours in healthy subjects significantly increased mean heart rate (+9 beats per minute; p < 0.010), systolic (+20 mmHg; p < 0.010) and diastolic blood pressure (+14 mmHg, p < 0.001) and plasma catecholamine levels [19]. In another study of flow-mediated endothelium-dependent vasodilatation of the brachial artery, significant decreases were found at one and two hours in those with impaired glucose tolerance or diabetes, but not in control subjects [20]. Other mechanisms of cell injury by hyperglycaemia include higher inflammatory response, higher circulating intercellular adhesion molecule (ICAM)-1 and increased production of superoxide radicals and other reactive oxygen species by oxidative stress [21]. Magnetic resonance studies [22] show that hyperglycemic or diabetic patients have greater myocardial infarct size [23], leading to impaired blood nutrition to the ischemic myocardial wall. Jensen et al [24] showed that hyperglycaemia at admission in STEMI patients who are successfully treated by percutaneous angioplasty is independently associated with the presence and extent of microvascular obstruction on contrast-enhanced magnetic resonance. Thus, microvascular obstruction as assessed by magnetic resonance may be a mechanism that relates ABG in acute STEMI to a worse outcome.

The results of admission blood glucose are concordant with others hyperglycaemia and insulin resistance evaluations in ACS patients. Sinnaeve et al [25] showed that both elevated fasting glucose and admission blood glucose worse in-hospital and 6-month mortality in ACS patients. Greater glucose level (fasting or admission), higher risk of in-hospital death, congestive heart failure, and cardiogenic shock. Su et al [26] studied glycaemic variability determined by a continuous 72-hours glucose monitoring system and the presence and severity of coronary artery disease in patients with type 2 diabetes mellitus. They found a correlation between mean amplitude of glycaemic excursions (difference between peaks and nadirs of glycaemia) and Gensini score (r = 0.277; p < 0.001), which assesses the severity of coronary artery disease: it grades narrowing of the lumen of the coronary artery and scores it as 1 for 1-25% narrowing, 2 for 26-50% narrowing, 4 for 51-75%, 8 for 76-90%, 16 for 91-99% and 32 for a completely occluded artery. Feinberg et al [27] compared 30-day and 1-year mortality in non-diabetic ACS patients, groups of patients with (359 patients) and without (701 patients) metabolic syndrome criteria. Hyperglycaemia increases 30-day mortality risk only in metabolic syndrome patients, but 1-year mortality was increased by hyperglycaemia in both groups (metabolic or non-metabolic syndrome patients). Or in impaired glucose tolerance ACS patients, long-term mortality is higher in patients with 2 h post-load hyperglycemia of ≥160 mg/dL, and equal to previously known diabetes [28]. Even in non diabetic patients with acute decompensated heart failure admissions without myocardial infarction, hyperglycaemia increases short and long-term mortality [29], maybe reflecting the same higher activation of the sympathetic system. In this setting, it is interesting the correlation of ABG with B-type brain natriuretic factor [30], a well-known heart failure prognostic factor.

Based on these results, how could we explain the absence of effect, or at least absence of linear effect, of hyperglycaemia in women’s SCA mortality? One possibility could be women’s comparative lower inflammatory response. Several studies show the influence of gender on inflammation. Schroder et al. [31] showed that women had lower TNF and higher IL-10 levels in sepsis. Oberholzer et al. [32] found that male trauma patients had higher IL-6 levels than women. Deshpande and colleagues [33] showed that estradiol attenuated the LPS-induced production of IL-1, IL-6, and TNF by macrophages and also decreased NFκB-binding activity. But some confounder factors can interfere in the results of studies of ACS patients. Women ACS patients are older than men in most of trials and databases, and older people also have higher hyperglycaemia, insulin resistance, erythrocyte sedimentation rate [34,35] like women. In other words, statistical adjustment for these confounder factors could not be enough.

Female STEMI adjusted in-hospital mortality persisted high, like other’s observations [9,10,36]. Vaccarino and co-workers [10] studied NRMI data and showed an increased risk in women for death even after coronary disease risk factor adjustment, except in patients over 75 years old, perhaps related to the comparative delay in hospital arrival, delay in diagnosis, and lower probability of receiving thrombolytic therapy. But 10 years later [12], the same group found that at ages 50 to 70 the higher adjusted mortality in women persisted, but not at other ages. So, they hypothesized that this was the result of greater awareness of heart disease in younger women in recent years or improved diagnosis or treatment of acute coronary syndromes [11]. This improvement in women’s cardiovascular mortality was observed in a study of a Brazilian population [37].

### Study limitations

This study assesses urban Brazilian ACS patients at a specialized cardiology center. Of STEMI patients, only 12 were treated with thrombolytics drugs, a situation not present in nonspecialized centers. We did not evaluate long-term outcomes, or assess risk scores like GRACE or TIMI.

## Conclusions

We conclude that age and ABG were independent risk factors for death in UA; age, lower ejection fraction, higher creatinine and ABG were independent risk factors for death in NSTEMI; and lower ejection fraction, higher creatinine and female gender were independent risk factors for death in STEMI. In global ACS patients, ABG influenced prognosis negatively in men, but this relation was not found in women, when hyperglycaemia was compared in the same gender.

## Competing interests

Conflicts of Interest and Source of Funding: Authors declare no conflicts of interest. This study was partially supported by FAPESP and CNPq.

## Authors’ contribution

JYT cared out paper conception, writing, data collection and statistical analysis. RBR, LCR, SDA participated in data collection. JAFR participated in data analysis and review process. APM carried out paper conception, data collection and analysis. All authors read and approved the final manuscript.
